# Critical Incidences in U.S. Health Care Systems Experienced by Undocumented Young Adults

**DOI:** 10.1089/heq.2020.0154

**Published:** 2021-09-08

**Authors:** Thomas Anthony Chávez, Selene C. Vences, Yazmin Irazoqui Ruiz, Josue De Luna Navarro, Felipe Rodriguez, Italia Aranda

**Affiliations:** ^1^ Department of Individual, Family, and Community Education, University of New Mexico (UNM), College of Education, Albuquerque, New Mexico, USA.; ^2^ UNM Transdisciplinary Research, Equity and Engagement Center for Advancing Behavioral Health (TREE), Albuquerque, New Mexico, USA.; ^3^ NM Dream Team, Albuquerque, New Mexico, USA.

**Keywords:** immigrant health, health disparities, health insurance, families

## Abstract

**Purpose:** Undocumented (“illegal immigrant”) young adults and families face many barriers when seeking health care, including discrimination, which contributes to health disparities. Using critical race theory, an investigation of experiences of health care among undocumented young adults was conducted to highlight their limitations to health care access.

**Methods:** Using a community-based participatory paradigm, a qualitative research approach was used to explore the experiences of 13 participants via a semistructured interview. Upon transcription and broad theme analysis conducted by the primary team members, a gathering of community experts was arranged. This gathering consisted of the primary team members as well as community young adults who identified as undocumented or of mixed-status families, in which all engaged in a collaborative analysis of themes and confirmation of corresponding illustrative data. Furthermore, community experts provided feedback on their insights for the necessary next steps.

**Results:** Through collaborative thematic analysis and confirmation of illustrative data, four themes emerged: (1) emotional and financial stress, (2) fear of exposure, (3) dependence on community health clinics, and (4) hospitals as a last resource.

**Conclusion:** Undocumented young adults and their families make great attempts to access health care, however, because of systemic barriers, they engage in strategies to preserve their safety in such attempts. Due to lack of insurance and financial strain, undocumented families depend on resources they most trust, typically health clinics. However, as many families depend on this resource, they may hinder efficiency in getting the specific help they need, especially if they do not have the capacity to adequately address medical issues that require immediate attention.

## Introduction

Latinx (this term is used throughout this article to be inclusive of gender-neutral and nonbinary identities in Latina/o communities) immigrants are frequently noted to have better health behaviors and health status than their U.S. counterparts; however, epidemiological data are incomplete as this population is not adequately surveyed.^1^ Nevertheless, health inequities among Latinx immigrant young adults and mixed-immigration status families (households where members hold different immigrant status) in the United States are evident and may have a basis in systemic barriers they face within the health care systems.^2^ Such barriers may include prejudice and discrimination based on immigrant status, race, language, and/or other stigmatized social identities.^2^ Other barriers include anti-immigrant policies, such as the Personal Responsibility and Work Opportunity Reconciliation Act (PRWORA), which created a two-tiered system of eligibility for immigrants of any background.

These acts also excluded undocumented immigrants (people who have immigrated from outside the United States and do not possess required visas and documentation to stay or have overstayed their temporary visa; also referred to as “illegal immigrants”) from receiving any form of public assistance, including Medicaid and Medicare.^3^ As a result, there are currently over 7.1 million noncitizens who do not have health insurance in the United States,^4^ therefore limiting their access to health care.

Workers in the United States largely receive their health care coverage from federal sponsorship or their employer, but when someone lives and works without documentation, they are not eligible for a social security number, which without they cannot apply for and obtain health insurance. Therefore, having a social security number is directly linked to having access to health care. When in need of medical services, a person or family member has to present to medical authorities without such a citizenship identity card, putting the person at risk of being found out and thus potentially being reported to legal authorities, such as Immigration and Customs Enforcement (ICE).

Further adding to this fear of being reported is the worry about out-of-pocket expenses and accumulated medical debt. Consequently, families are forced to endure illnesses that can often lead to even more serious issues, which could have otherwise been addressed preventatively. The reality of living undocumented and not having health insurance is detrimental to the undocumented and mixed-status community's overall health.^1^

Lawmakers often ignore the global health statistics among undocumented communities and deficiencies in the U.S. health care systems. The United States spends two times more than other developed countries on health care per capita.^1^ Yet, in terms of health care quality, the United States does not compare with other countries spending half as much.^1^ One of the biggest concerns in the health care system is accessibility, especially among the low-income, undocumented immigrants of color. The Affordable Care Act (ACA) has extended health insurance coverage and has shown to increase health care access among communities of color,^5^ however, undocumented communities are excluded from such benefits as they are ineligible to receive health insurance through the ACA. This leaves 3.3% of the U.S. immigrant population (∼10.7 million) without insurance coverage, including U.S.-born children with an undocumented parent.^6^

There are no federal or state laws that prevent undocumented immigrants from obtaining and accessing medical care. There are also no federal or state laws that restrict physicians in treating undocumented individuals.^7^ However, while health insurance coverage or eligibility for federal programs is not equivalent to physical access, health insurance is the primary determinant of actually accessing health care.^8^

The only access to health care immigrants is guaranteed emergency medical care provided through the 1986 Emergency Medical Treatment and Active Labor Act (EMTALA).^9^ As such, community health clinics and federally qualified health centers are where undocumented immigrants receive most of their health care.^10^ Immigrant populations can receive services, such as primary medical care, dental care, and behavioral health, at these nonprofit health care centers that now exist in urban, suburban, and rural areas.

The current article presents findings from a larger study on the impact of prejudice and discrimination reported by undocumented young adults in health and educational institutions in a largely rural state. We present the individual experiences of critical health care incidents among undocumented young adults within their family context who may be of mixed immigration status or further delineated as “mixed-status” in this article.

Mixed-status is a term used to describe a household where there are individual family members of a household who have different types of immigration statuses, such as undocumented, legal permanent resident, and DACA (Deferred Action for Childhood Arrivals) recipient. In 2012, President Obama issued the DACA executive order after the Development, Relief and Education for Alien Minors (DREAM) Act. The DREAM act allowed immigrant students, who primarily lived in the United States most of their lives, temporary legal status and future eligibility for U.S. citizenship if they went to college or joined the U.S. military.

A critical perspective was maintained throughout the investigation due to the investigators' observations that social, cultural, and political factors are intricately involved in the lives of undocumented immigrant identity and health. Specifically, the critical race theory (CRT) provided the investigators a lens through which to center Latinx young adults' experiences of race and color-based prejudice and discrimination, social dynamics, and marginalization for which to inform policies on immigration health reform for this community.^11^

## Methods

### Establishing a community-based participatory research team

Funding for this study was provided by the University of New Mexico Transdisciplinary Research on Equity and Engagement and approval was provided by the University of New Mexico Institutional Review Board (UNM IRB#: 947739-4). The study occurred during the period 2016–2019. A critical goal of this qualitative investigation was to use a community-based participatory research (CBPR) approach increasingly used in health disparity research to address issues of inequities within research and misrepresentations often made by community outsiders.^12^ Using qualitative methods allowed us to highlight the voice of an otherwise silenced population, for example, the Latinx undocumented immigrant community.^13^ A CBPR approach allowed us to be driven by the community whose knowledge, voice, and consent were promoted in every step of the research process to collaboratively work toward an agenda for social change in immigrant health equity.^12^ As such, it was important to develop a working alliance that prioritized genuine relationships, collaboration, and shared power between the academic setting and the community. Each entity equally partook in every step, including the assessment of community needs, planning of investigative processes, research design, analysis, and dissemination.

To ensure adherence to aspects of CBPR in the entire investigative process, a team was assembled to include members from the undocumented community. Five mixed-status, college-aged young adults who were organizers of local chapters of the national United We Dream (UWD) organization collaborated with the principal investigator. Each team member was proficient in Spanish/English bilingualism and had scholarly backgrounds in various disciplines, including psychology, counseling, medicine, engineering, and law. The primary investigator identified as Latinx, however, not part of the undocumented immigrant community and depended, with cultural humility, on the coinvestigators for prioritized information of undocumented immigrant experiences. Team members identified as cisgender men and women with one being part of the queer community. The larger undocumented young adult community who participated in this CBPR project provided valuable input into other diverse and intersectional identities as detailed below.

### Demographics

The 13 participants included state-wide young adults from a U.S. southwestern state, recruited via social media, flyers, e-mails, and phone calls to the UWD local chapter listservs. Inclusion criteria for participation required being age 18 or older and identification as a member of the Latinx undocumented immigrant community, that is, a DREAMER, DACA, etc., or having family members who identified as such. All the qualifying participants included young adults from university satellite urban, semirural, and rural college settings who provided participation consent in preferred English or Spanish formats. Table 1 displays the demographic information of research participants.

**Table 1.** Demographic Information Collected from Research Participants

Social identity	No. (%)	Immigration status	No. (%)
DREAMer	6 (46)	DACAmented	6 (46)
Undocumented	8 (62)	Undocumented	3 (23)
Undocu-Queer	2 (15)	Lawful Permanent Resident	1 (8)
Nationality	5 (38)	U.S. Citizen	2 (15)
Latino/a	7 (54)		

Gender identity	No. (%)	Primary language	No. (%)
Female	8 (62)	English	13 (100)
Male	5 (38)	Spanish	0 (0)

LGBTQIA+	No. (%)	Had health insurance	No. (%)
Yes	2 (15)	No	9 (69)
No	11 (85)	Yes	4 (31)

LGBTQIA+, Lesbian, Gay, Bisexual, Trans, Queer, Intersex, Asexual, and other sexual minorities.

Research interview protocol question examples included “Can you share about experiences where you have refrained from accessing any health care services because of particular fears” and “can you share a time you have felt like you were discriminated against due to your color of skin or immigration status while accessing health care?” “Data adequacy” or saturation was reached at a total of 13 interviews in which no new data emerged.^14^ Each interview was digitally recorded, transcribed in its original language (English, Spanish, or bilingual), deidentified, confidentiality stored, organized, and analyzed using qualitative analysis software (NVivo 12).

### Community experts

Community experts, consisting of ∼15 nonresearch participant community members per group, were recruited through the local UWD chapters. Gatherings of two community expert groups were held in two chapters, at two separate times. The intention of the community expert gatherings was to confirm data and thematic analysis already conducted by the primary research team.

A first step in this process was to orient the community experts to the research purpose and sensitize them to a critical perspective. This was completed by guiding the attendees through an “Undoing Racism” activity titled the “Foot of Oppression,” adapted from the People's Institute for Survival and Beyond.^15^ In this process, the community experts were introduced to the concept of oppression, which many were already familiar with. A large circle on a flipchart was drawn by the facilitator, representing a community, in which community experts instructed the facilitators what to draw or write. The community experts were asked to identify the particular situations and institutions that contributed to oppression in their communities, emphasizing the institution of health and health care.

Upon completion of the “Foot of Oppression” activity, the facilitators posted large sheets of paper around the room, which had prewritten themes that were gathered in the last iteration of the primary team data analysis. The community experts were provided precut pieces of paper that had the deidentified quotes from the individual participant interviews. The community experts were directed to tape the quotes as they saw fit on the theme titled large papers around the room. During this process, community experts freely dialogue thoughts on the quotes with each other.

If a new theme emerged, a new large piece of paper was posted on the wall in the room to have related quotes taped onto. This process generated dialogue between community experts and the primary team members, further informing the intricacies of the phenomena under investigation. This assisted the primary research team to collapse themes through follow-up discussions. While the community experts provided the confirmation and final decision for which themes to best be discussed in this article, the meeting discussions and input were not recorded, and as such not used as data.

### Analysis

The primary research team met biweekly over 3 months to share our experiences with the population under investigation and collaboratively make decisions about themes and interpretation of data. All data were analyzed in Spanish, English, or bilingual forms to maintain linguistic and cultural meanings.

The primary six team members discussed insights gathered from the thematic analytical process of participant thoughts and feelings indicated by thematic analysis and tenets of CRT.^11,15,16^ Ford and Airhihenbuwa^11^ explains that using CRT sensitizes researchers to the impact race and racism have on the health and well-being of racialized and marginalized individuals and groups. Furthermore, these experiences produce knowledge, critical consciousness, and a deep understanding of institutional and systemic health inequities, which may not be accessible to investigators who may not identify as a member of the community being investigated.

CRT was thus applied in this study at every step, from the initial reading of transcripts, definition of codes, development and interpretation of themes, to article development. Thematic analysis, according to Braun and Clarke,^16^ is flexible in data identification, analysis, and report of patterns in qualitative data.^16^ This method allowed the coinvestigators to collaboratively develop codes that led to prominent inductive and underlying or latent ideas, assumptions, and ideologies of themes.

Maintaining the CRT framework, four rounds of thematic analysis were conducted.^11,16^ First, individual case-level thematic analysis occurred, which entailed notation and memoing of meaning patterns in the first reading of individual transcripts. This was followed by another reading to generate initial codes (e.g., “fear of being reported”). Further iterative readings then consisted of collapsing codes and searching for themes, reviewing themes, defining and naming themes, and providing a within-case report to team members. In the second round, a collaborative thematic development led to the development of a codebook. Third, a final round of collaborative thematic coding of all data was completed with the codebook. Lastly, the significance and trends of themes in the data were discussed.

Confirmation and member checking of themes and corresponding participant quotes were made in collaboration with the community experts. This aspect further followed the tenets of a community-based participatory approach, highlighting the knowledge of community members necessary for community-responsive investigation and potential nuanced interventions.^17^

## Results

Thematic analysis with a guiding lens of CRT resulted in four major intersecting themes: (1) the emotional and financial stress of lacking health insurance, (2) fear of exposing one's immigration status to a health care institution, (3) dependence on community health clinics, and (4) seeing hospitals as a last resource. We provide example quotes of the themes in Table 2, however, only highlighting a few in the following text. Such quotes represent the complex situations in which undocumented and mixed-status families find themselves in the process of accessing health care. Pseudonyms were assigned to protect the identities of all the participants.

**Table 2.** Illustrative Quotes of Emerging Themes

Emotional and financial stress of lacking health insurance	Fear of exposing one's immigration status to a health care institution
Every year my sister [who is undocumented] used to get these huge fevers!…I remember one of the nights that my sister got very ill. She got admitted to the hospital and I remember seeing her while she slept, all sweating…I remember asking myself “What is going to happen to my sister?”…[After that] my sister could never go to the bi-monthly physicals [because of her lack of insurance]…[the doctors] detected a tumor in her brain and they has to do a surgery and because she can't get insurance it would cost us $10,000…My sister had to go to get financial help like five times to try to get a payment plan…it really sucks to see my sister in surgery and be thinking [about]how we are going to pay for it (America, 19 years old).	Once someone gets health insurance it's another whole world when you go to the doctor because I think my parents didn't take us to like the dentist or the doctor because we didn't have health insurance and my parents didn't speak English. So they couldn't…like there were things to help us out but my parents just didn't know how to ask for them since they didn't know the language…Growing up we never seeked any help like that and also my parents were really afraid of asking for help because “la migra” (ICE) was going to get us (Nadine, 31 years old).
I have never requested or been able to [access community resources and hospitals]. I mean the only times has been like…hum… going to the doctors to “La Familia” [Clinic] because of the discount but that's it… if you don't have a social security number or health insurance it's not the same… that's what I have discovered. Once someone gets a health insurance it's another whole world when you go to the doctor because when you don't have a social security number or health insurance they don't do so much for you but once you have a health insurance they would do every single test they can to find whatever you have (Juice, 31).	My mom would avoid any sort of public assistance. The only time I and even my mom would go to the hospital was if it was an emergency[…] most people that are not undocumented, when they feel bad they go to the hospital and they schedule an appointment with a doctor… that was not the case for me and my family… we were just told to rest and have some remedy from my grandma or something like that… but only a few times we had to go to the hospital … we wanted to avoid it as much as possible though[…] in general I would say that as an undocumented person you fear that (Raul, 29 years old).

Dependence on community health clinics	Seeing hospitals as the last resource
I do remember going to the hospital once but it was because I broke my nose…well someone broke my nose…[In the past], I had friends that needed an ambulance and the ambulance actually came for them but that bill was…and friends who were also undocumented actually…and the bill was a $5, 000 bill just for an ambulance. SO, knowing about the bills, [when that person broke my nose], I decided to get up the next say that I went to a local clinic to get and x-ray and MRI, actually. If I was [an]adult, that MRI would have cost me $5,000 plus [the] ambulance that would be $10,000, plus service for re-breaking my nose and putting it back into place was another charge (Juan, 28 years old).	[My dad] got very ill but it wasn't until they forced him to take a week off so he could rest, then he did it…like people had to force him to take care of himself. Otherwise, he would continue going to work…I think that's true as a community though…we turn to go to the hospital until we are dying or like when we really need to go and we don't know of resources…So, it's like we don't go because we don't know of resources and when we do know we don't go because we don't have money for it (Luz, 19 years old).
We had access to community clinics so like… the first choice clinics. Yeah I think it was the first place we visited because for school you had to be vaccinated and they wanted to make sure you had the shot record and all that so that was the first place… It was actually one of the first and accessible places that we had because we couldn't go to the hospital because it was really expensive and all that (Tina, 23 years old).	…if we went to the doctor we went to the emergency room. We didn't have like a regular doctor we would go to… and we didn't know of any clinics that would take you if you didn't have a social security number … so, we knew we could go to the ER but I remember at that time my mom would give a… a fake SSN [Social Security Number] but like…. It wasn't until later in New Mexico that I realized we didn't have to have a SSN to get emergency care[…] My brothers had regular doctors…but like my brothers are US citizens! (laughs) But… I don't remember how often we would go, but my mom has suffered from hypertension and like… Cholesterol and things like that… so there was no other option, we would have to go to the ER (Adela, 24 years old).

The voice of each participant has not been changed and kept in close linguistic interpretation as possible. Noted are the emotional, social, cultural, political, economic, and structural/institutional challenges faced by undocumented immigrant youth and their families.

### The emotional and financial stress of lacking health insurance

Illustrating the *Emotional and Financial Stress of Lacking Health Insurance* theme, 19-year-old America discussed how her undocumented sister struggled with navigating the health care system, but fortunately had a documented extended family member who worked as a nurse at the hospital and helped them through her medical concern. America shared that growing up in a mixed-status family meant that all family members encountered health care challenges due to not being insured. Not having health insurance meant that the family would have to pay out-of-pocket. Furthermore, this deterred her sister from follow-up appointments despite the serious medical condition of possibly having a brain tumor. America's sister tried multiple times to get financial assistance, but this, too, was a systemic barrier.

Other participants in the study also expressed that if assistance was sought, families feared being accused of “abusing the system” and thus threatening “eligibility to become legal residents” (Raul, age 29). These interactions, often had the tone of anti-immigrant or race-based biases. America noted, for example, the delay for financial assistance, “trying 5 times” to get financial help, due to being undocumented.

For undocumented families in this study, systemic barriers contributed to overwhelming emotional distress due to the financial burden and life-threatening health concerns of a loved one. Even seeking out assistance placed one at risk as one had to disclose sensitive information in the process, including documentation status. Nadine (31 years old) further explained in her interview, like other participants, that this also had implications of risking family member's work toward legal citizenship or naturalization.

### Fear of exposing one's immigration status to a health care institution

Being undocumented in the United States involved the fear of exposing one's immigration status to institutions. Most fears came from the idea that one will have immigration officers called on them and thus leading to one's deportation. The quote by Nadine represents the *Fear of Exposing One's Immigration Status to a Healthcare Institution* theme. It illustrates, as in the first theme, how the lack of insurance deters parents from accessing services for their children. Also, Nadine highlights how language can be a barrier to requesting what one needs as well as a risk for being exposed as undocumented simply by speaking Spanish. As such, disclosure of an undocumented immigration status in whatever language produced perpetual fear of being deported.

### Dependence on community health clinics

Many of the participants expressed that they or family members would avoid going to hospitals and some even stated depending on natural remedies (America and Raul). If going for services, they each had *Dependence on Community Clinics* for health needs that natural home remedies could not take care of. In the case of 28-year-old Juan, even with a broken nose when he was younger, he avoided going to the hospital to also avoid the financial burdens of high medical bills. He had to negotiate this as a young person even knowing that going to the clinic the next day would delay appropriate care for his condition. Juan was conscious of the fact that the clinic provided young patients, as he stated, “a discount” and that it would cost his family more going to the hospital.

### Seeing hospitals as the last resource

Unfortunately, the combination of the lack of health insurance and fear of exposing one's immigration status led undocumented and mixed-status families to *Seeing Hospitals as the Last Resource* when other home and community resources were not able to be used. For example, one participant shared “…we didn't know of any clinics that would take you if you didn't have a social security number…so, we knew we could go to the ER…” (Adala, age 24), which not all families know is possible. Luz, age 19, described how her father would endure illness and push himself through work rather than seek medical attention. This occurred despite the given time off for self-care. Luz observed this common theme in her community in which only if it were a life or death situation would community members go to the hospital.

Once again, financial resources were inaccessible to pay for medical costs. Utilization of a hospital as a last resource because of financial strain was common among our participants and jeopardized the health of our participants and families. Other times there was a fear of not being adequately attended to due to their undocumented status as in the case of a 31-year-old participant named Juice who shared that “we went to a hospital first but they didn't want to treat [my mom for H1N1] and then we went to another one and she got admitted that day and actually went into a coma.”

Overwhelmingly, participants and community experts were concerned with the heightened health risks their families endured. Furthermore, they also noted microaggressions and intentional acts of neglect based on anti-immigrant sentiments and race or skin color. Because the participants and experts were involved with UWD advocacy and organizing efforts, current U.S. sociopolitical issues surrounding immigration and policies became a critical part of the dialogue and the subsequent push for social change. Specifically, it was discussed that health care insurance is open and accessible to undocumented families and that there has be an increase of built community clinics in low-income communities.

## Discussion

The voices of Latinx undocumented immigrant young adults in this study illustrate a profound struggle as they and their families navigate health care services. The inaccessibility of health insurance encountered by undocumented immigrant communities must be addressed at many institutional levels as without attention, it compromises their health, life expectancy, and mortality.^18,19^

The results of this study coincide with other studies that demonstrate how low utilization of hospital and clinic services is rooted in inaccessible health insurance, enduring financial concerns, fear of exposing one immigration's status, experiences of delayed services, and language barriers.^20^ While some participants in this study mentioned experiences of anti-immigrant sentiment, microaggressions, and racism while trying to access care as another reason for underutilization of services, there is still a great need for a better understanding of how it impacts immigrant health.^21^

Fear of deportation after disclosure of immigration status is often cited as a barrier to accessing health services and causes much emotional distress.^22^ Studies have shown that Latinx immigrant families carry great mistrust of health care systems, leading them to avoid much-needed services, and thus end up enduring much pain and suffering.^23^ And while the use of home remedies or traditional medicine, common among many Latinx populations, can be useful as preventative measures, it should not be used solely because of health care barriers.^21^

This is concerning when societies as a whole are at great risk for health issues such as the H1N1 epidemic, as mentioned by one participant. These concerns are further exacerbated with the COVID-19 pandemic, which disproportionately impacts the lives of Latinx undocumented immigrants.^23^

Some health care institutions recognize the need to support policies and programs as more evidence grows that demonstrates the impact on Latinx health disparities.^24,25^ However, policymakers must work to pass legislation that will allow undocumented families to have diverse avenues to health insurance. Providing undocumented individuals access to health coverage is not a new idea in the United States. For example, California instituted the Medi-Cal program in response to the noted large population of undocumented and uninsured families in the state. Despite this program being responsive to the undocumented immigrant issue, it is noted that there were more barriers to overcome within the program, For example, an undocumented individual has to earn 138% above the federal poverty level to be eligible as indicated in this program.^14^

Data consistently show how access to health care insurances increases the overall community's health and improves an individual's health care outcomes.^26^ Other policy-level initiatives, such as utilizing driver's licenses to access health care rather than a social security number is something to be seriously considered,^19^ although allowance for undocumented individuals to obtain a driver's license varies from state to state. Finally, there can be intervention efforts to help Latinx immigrant families navigate health care resources and be informed of their health care rights and eligibilities, which often do not exist in communities where immigrants make up a portion of the population.^22^ Such resources are increasingly scarce when we consider the lives of immigrants in rural communities.

The narratives illustrated in this work are remarkably noted in Aguilar's Undocumented Critical Theory,^27^ in which fear and liminal uncertainty are experienced daily, not knowing where they stand in the American society. Families and communities serve as resources of resiliency for each generation to move forward. Aguilar emphasizes how the “documented/undocumented” binary no longer suffices to fully understand the diverse inequities within mixed-status communities and the diversity within needs to be intricately investigated.

Nevertheless, it is critical to dismantle barriers to health that stem from anti-immigrant sentiment and racism, thereby improving health equity for undocumented and mixed-status families. Hacker et al. recommended that to do so, U.S. health care systems need to emphasize (1) advocacy for health care access and rights to citizenship, (2) an allowance for all to have access to state-funded insurance, (3) an expansion of health service capacity for this population, (4) training of providers, and (5) culturally and linguistically responsive outreach and education toward helping undocumented families better navigate health care.^1^

## Conclusion

This study explored how undocumented young adults and their families make great attempts to access health care. Systemic barriers force these families to engage in strategies to preserve their safety from being deported in the process of such attempts. Due to lack of insurance and financial strain, undocumented families depend on resources they most trust, typically community clinics. As many families depend on this resource, they may hinder adequate and efficient help, especially if not having the capacity to address medical issues that require serious and immediate attention. To break down the barriers faced by undocumented immigrant families, great attention is critical in terms of advocacy for policy change and efforts in addressing anti-immigrant and racist sentiments that exist within the health care system at interpersonal and structural/institutional levels.

## Abbreviations Used

ACA: Affordable Care Act
DACA: Deferred Action for Childhood Arrivals
DREAM: Development, Relief and Education for Alien Minors
CBPR: community-based participatory research
CRT: critical race theory
UWD: United We Dream

## References

[1] Escarce JJ, Morales LS, Rumbaut RG. The health status and health behaviors of Hispanics. In: Hispanics and the Future of America. Edited by Tienda M, Mitchell F. Washington, DC: National Academies Press, 2006, pp. 362–409. 20669436

[2] Hacker K, Anies M, Folb BL, et al. Barriers to health care for undocumented immigrants: a literature review. Risk Manag Healthc Policy. 2015;8:175–183. 26586971 10.2147/RMHP.S70173 PMC4634824

[3] Perreira KM, Pedroza JM. Policies of exclusion: implications for the health of immigrants and their children. Annu Rev Public Health. 2019;40:147–166. 30601722 10.1146/annurev-publhealth-040218-044115 PMC6494096

[4] Budiman A, Tamir C, Mora L, et al. (2020, August 20). Immigrants in America: current data and demographics. Available at https://www.pewresearch.org/hispanic/2020/08/20/facts-on-u-s-immigrants-current-data Accessed October 13, 2020.

[5] Glied SA, Ma S, Borja A. Effect of the Affordable Care Act on health care access. Available at https://www.commonwealthfund.org/sites/default/files/documents/___media_files_publications_issue_brief_2017_may_glied_effect_of_aca_on_hlt_care_access_ib.pdf Accessed October 10, 2020. 28574234

[6] López G, Bialik K, Radford J. (2018, November 30). Key findings about U.S. immigrants. Available at http://www.pewresearch.org/fact-tank/2018/11/30/key-findings-about-u-s-immigrants Accessed October 12, 2020.

[7] Ambegaokar S. Opportunities for maximizing revenue and access to care for immigrant populations. n.d. Available at https://www.astho.org/Public-Policy/Public-Health-Law/Access-to-Care-for-Immigrant-Populations-Overview Accessed October 10, 2020.

[8] Llano R. Immigrant and barriers to healthcare: comparing policies in the United States and the United. 2011. Available at https://web.stanford.edu/group/sjph/cgi-bin/sjphsite/immigrants-and-barriers-to-healthcare-comparing-policies-in-the-united-states-and-the-united-kingdom Accessed October 10, 2020.

[9] Gusmano MK. Undocumented immigrants in the United States: U.S. health policy and access to care. The Hastings Center. 2015. Available at http://undocumentedpatients.org/issuebrief/health-policy-and-access-to-care Accessed October 10, 2020.

[10] Wright B, Ricketts TC. When patients govern: federal grant funding and uncompensated care at Federally Qualified Health Centers. J Health Care Poor Underserved. 2013;24:954–967. 23728059 10.1353/hpu.2013.0068 PMC5590367

[11] Ford CL, Airhihenbuwa CO. Critical race theory, race equity, and public health: toward antiracism praxis. Am J Public Health. 2010;100 Suppl (Suppl 1):S30–S35. 20147679 10.2105/AJPH.2009.171058 PMC2837428

[12] Wallerstein NB, Duran B. Using community-based participatory research to address health disparities. Health Promot Pract. 2006;7:312–323. 16760238 10.1177/1524839906289376

[13] Ojeda L, Flores LY, Meza RR, et al. Culturally competent qualitative research with Latino immigrants. Hisp J Behav Sci. 2011;33:184–203.

[14] Morse JM. The significance of saturation. Qual Health Res. 1995;5:147–149.

[15] Undoing racism, The People's Institute for survival and beyond. 2020. Available at https://www.pisab.org Accessed October 10, 2020.

[16] Braun V, Clarke V. Using thematic analysis in psychology. Qual Res Psychol. 2006;3:77–101.

[17] Health Sciences Center, Center for Participatory Research. 2020. Available at https://cpr.unm.edu/research-projects/cbpr-project/cbpr-model.html Accessed October 10, 2020.

[18] Pérez-Escamilla R, Garcia J, Song D. Health care access among Hispanic immigrants:¿ Alguien está escuchando?[Is anybody listening?]. NAPA Bull. 2010;34:47–67. 21116464 10.1111/j.1556-4797.2010.01051.x PMC2992323

[19] Singh GK, Rodriguez-Lainz A, Kogan MD. Immigrant health inequalities in the United States: use of eight major national data systems. Sci World J. 2013;75:2099–2106. 10.1155/2013/512313 PMC3826317 24288488

[20] Ransford HE, Carrillo FR, Rivera Y. Health care-seeking among Latino immigrants: blocked access, use of traditional medicine, and the role of religion. J Health Care Poor Underserved. 2010;21:862–878. 20693732 10.1353/hpu.0.0348

[21] Viruell-Fuentes EA, Miranda PY, Abdulrahim S. More than culture: structural racism, intersectionality theory, and immigrant health. Soc Sci Med. 2012;75:2099–2106. 22386617 10.1016/j.socscimed.2011.12.037

[22] Rhodes SD, Mann L, Simán FM, et al. The impact of local immigration enforcement policies on the health of immigrant Hispanics/Latinos in the United States. Am J Public Health. 2015;105:329–337. 25521886 10.2105/AJPH.2014.302218 PMC4318326

[23] Calderone JR, Abel AM, Rosenbaum E. COVID-19 and undocumented immigrants. Stronger Together. 2020;37:1–3.

[24] Stone LC, Steimel L, Vasquez-Guzman E, et al. The potential conflict between policy and ethics in caring for undocumented immigrants at academic health centers. Acad Med. 2014;89:536. 24556759 10.1097/ACM.0000000000000187 PMC6522141

[25] Philbin MM, Flake M, Hatzenbuehler ML, et al. State-level immigration and immigrant-focused policies as drivers of Latino health disparities in the United States. Soc Sci Med. 2018;199:29–38. 28410759 10.1016/j.socscimed.2017.04.007 PMC5632125

[26] McConville S, Hill L, Ugo I, et al. Health Coverage and Care for Undocumented Immigrants. San Francisco, CA: Public Policy Institute of California, 2015, pp. 1–11.

[27] Aguilar C. Undocumented critical theory. Cult Stud↔Crit Methodol. 2019;19:152–160.

